# Multifaceted Roles of the E3 Ubiquitin Ligase RING Finger Protein 115 in Immunity and Diseases

**DOI:** 10.3389/fimmu.2022.936579

**Published:** 2022-06-30

**Authors:** Mei-Xia Wang, Tianzi Liuyu, Zhi-dong Zhang

**Affiliations:** ^1^ The Executive Master of Business Administration (EMBA) Program, School of Management, Fudan University, Shanghai, China; ^2^ Department of Gastrointestinal Surgery, Medical Research Institute, Zhongnan Hospital of Wuhan University, Wuhan, China

**Keywords:** RNF115, antiviral immunity, autoimmunity, cancers, DNA damage repair

## Abstract

Ubiquitination is a post-translational modification that plays essential roles in various physiological and pathological processes. Protein ubiquitination depends on E3 ubiquitin ligases that catalyze the conjugation of ubiquitin molecules on lysine residues of targeted substrates. RING finger protein 115 (RNF115), also known as breast cancer associated gene 2 (BCA2) and Rab7-interacting RING finger protein (Rabring7), has been identified as a highly expressed protein in breast cancer cells and tissues. Later, it has been demonstrated that RNF115 catalyzes ubiquitination of a series of proteins to modulate a number of signaling pathways, and thereby regulates viral infections, autoimmunity, cell proliferation and death and tumorigenesis. In this review, we introduce the identification, expression and activity regulation of RNF115, summarize the substrates and functions of RNF115 in different pathways, and discuss the roles of RNF115 as a biomarker or therapeutic target in diseases.

## Introduction

Ubiquitin is a 76-amino-acid highly conserved protein that is covalently conjugated to and commonly forms polyubiquitin chains on the lysine residues of protein substrates (1). Such a modification by ubiquitin is known as ubiquitination that is sequentially mediated by a three-enzyme cascade consisting of E1 ubiquitin-activating enzyme, E2 ubiquitin-conjugating enzyme and E3 ubiquitin ligase. E1 activates the C terminal carboxyl group of ubiquitin and transfers it to the active site cysteine of E2 to form a thioester bond. Subsequently, E3 ubiquitin ligases catalyze the transfer of the C terminal carboxyl group of ubiquitin to the ϵ-amino group of a lysine residue in the substrate to form an iso-peptide bond (2). Consecutively, more ubiquitin molecules can be linked to one another to form a K-X-linked or linear polyubiquitin chain (X represents the lysine residue on one ubiquitin whose ϵ-amino group is linked to the C terminal carboxyl group of another) that determines the function and fate of substrate proteins. For example, K48- or K11-linked polyubiquitin modifications often dictate proteins for proteasome degradation (3), whereas K63- or K27-linked polyubiquitin modifications serve as signaling platforms for protein activation and recruitment of downstream proteins (4).

The E3 ubiquitin ligases control the efficiency, substrate specificity and ubiquitin linkage specificity and therefore constitute the core of the ubiquitination reaction (5). To exploit the functionality of protein ubiquitination, the human genome encodes more than 600 E3 ubiquitin ligases that are classified into five families: the really interesting new gene (RING) finger proteins (6), the homologous to E6- anaphase-promoting complex (APC) terminus (HECT) proteins (7), the RING-in-between-RING (RBR) proteins (8), the U-box domain-containing proteins (9), and the plant homeodomain (PHD) finger proteins (10). The RING finger E3 ligases are the largest family and function alone or as a component of the cullin-RING ligase (CRL) complex to catalyze the ubiquitination of substrates (11). By catalyzing ubiquitination of substrates, RING finger proteins exert various cellular and physiological functions including immune responses, development and tumorigenesis. For example, the RING finger protein MDM2 targets p53 for degradation in human cancers (12), and another RING finger protein Cbl regulates immune system development and function by catalyzing ubiquitination of a number of substrates including CD3, FLT3, and IRF4 (13). It is of great interest and importance to characterize the functions of RING finger E3 ubiquitin ligases by identifying their targets and regulatory mechanisms.

RNF115 which is also known as Rabing7 (Rab7-interacting RING finger protein), BCA2 (breast cancer-associated gene 2) and ZNF364 (Zinc finger domain-containing protein 364) belongs to the RING finger family and functions independent of the CRL system to catalyze polyubiquitination of various substrates (14, 15). The available studies on RNF115 have demonstrated that RNF115 functions dependently on its E3 ligase activity, indicating that the ubiquitination of substrates mediates the functions of RNF115. In this review, we first introduce the identification and biochemistry of RNF115, including the expression, structure, and regulatory mechanisms. Then, we summarize the substrates and signaling pathways that are regulated by RNF115. Finally, we review the physiological and pathological functions of RNF115 and make perspectives on the future research about RNF115.

## The Identification and Biochemistry of RNF115

### The Identification of RNF115

By using a subtractive cloning technique, Burger et al. have obtained hundreds of cDNA clones that encode genes differentially expressed in breast cancer cell lines compared to normal breast cell lines (16, 17). Subsequently, a 325 bp partial cDNA sequence of the *RNF115* gene originally termed T3A12 was identified in the cDNA library (16). Later, the full-length sequence of *RNF115* mRNA was cloned and named as BCA2 that was identical to the hypothetical zinc finger protein ZNF364 (GenBank accession no. NM_014455) (14). Earlier than this study, another group identified mouse RNF115 as a Rab7-associated protein by yeast two-hybrid assays with Rab7 as the bait to screen Rab7-interacting proteins from a murine immature B cell cDNA library. It was found that such a protein was identical to a clone encoding ZNF364 (GenBank accession no. MN_026406) and named as Rabring7 (Rab7-interacting RING finger protein) (15).

**Figure 1:**
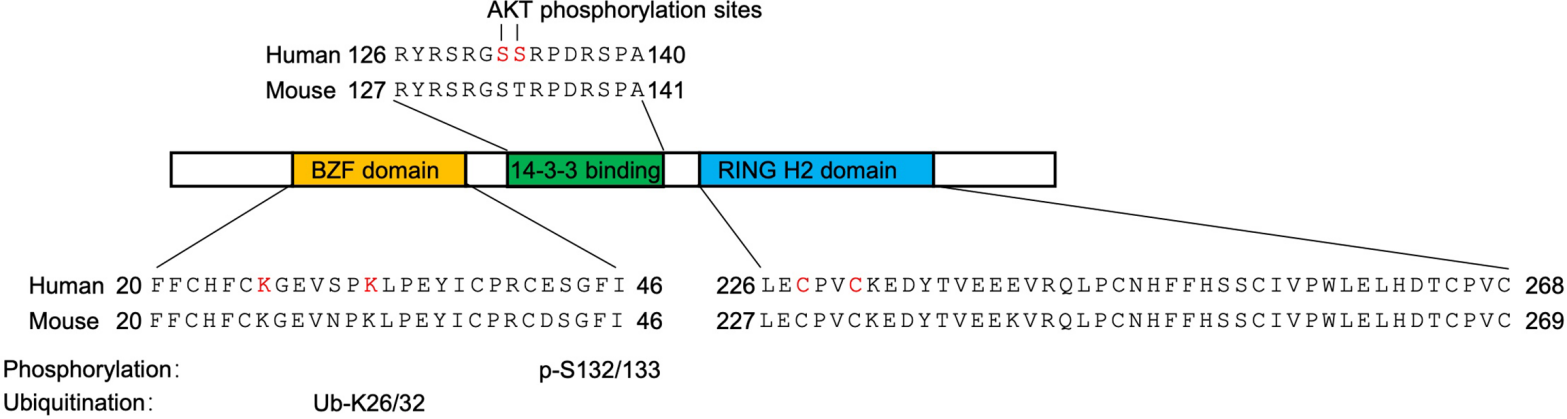
Conserved domains of RNF115. The BZF domain (yellow) binds ubiquitin and is the site of RNF115 auto-ubiquitination (ubiquitinated lysine residues are shown in red). The 14-3-3 binding domain (green) is site of RNF115-14-3-3 interaction (AKT phosphorylation residues are shown in red). The RING H2 domain (blue) is the site of RNF115 autoubiquitination. The E3 ligase activity residues were shown in red.

Soon after the release of the complete human genome sequence, the human *RNF115* gene has been mapped to chromosome 1q21.1 where frequent chromosomal imbalances have been reported in breast cancer (18, 19). The *RNF115* gene consists of 9 exons spanning 88,228 bp of genomic DNA. It is predicted that there are 6 isoforms of mature *RNF115* mRNA and the longest one is 9,135 nt with an open reading frame of 915 nt encoding a 304 aa polypeptide. The mouse *Rnf115* gene is located at chromosome 3 and similarly consists of 9 exons spanning 63,545 bp of genomic DNA. It is predicted that there are three different isoforms and the longest one is 2265 nt with an open reading frame of 918 nt encoding a 305 aa polypeptide. More recently, the *RNF115* genes of large yellow croaker and feline have been cloned to encode proteins consisting of 295 aa and 305 aa, respectively (20, 21). Bioinformatics studies suggest that RNF115 contains an N-terminal C2/C2 ubiquitin-binding zinc finger (BCA2 Zinc-Finger, BZF) domain and a C-terminal RING-H2 domain that are evolutionarily conserved from nematodes to mammals (22–24) (**Figure 1**). Results from Northern blot and immunoblot assays have demonstrated that RNF115 is widely expressed in various human and mouse tissues including heart, lung, liver and testis, in cell lines such as MCF7, MDA-MB-468, WEHI231, MDCK and BHK cells, and in primary murine cells including mouse embryonic fibroblasts (MEFs), bone marrow-derived dendritic cells (BMDCs), bone marrow-derived macrophage (BMDMs) and peripheral blood monocyte-derived DCs (14, 15, 25). Collectively, these early studies show that RNF115 is a zinc finger and RING finger domain-containing protein that is widely expressed in various tissues and cells and upregulated in breast tumors.

### The E3 Ligase Activity of RNF115

Because RNF115 contains a typical RING finger domain, the E3 ligase activity of RNF115 has been extensively studied. It has been observed that RNF115 purified by affinity chromatography from the baculovirus expression system in Sf9 cells efficiently induces the formation of a high molecular weight smear on the anti-ubiquitin immunoblots in the presence of ubiquitin, E1 and E2s such as Ubc2, Ubc4, UbcH5 and UbcH6 (26). In addition, the endogenous BCA2 purified from MCF7 and T47D breast cancer cell lines also promotes the formation of ubiquitin smears when incubated with recombinant UbcH5b, ubiquitin, and E1 (14). Such ubiquitin smears are completely abolished when the C228/231 of hRNF115 (and the C229 of mRNF115) are mutated into A (14, 26, 27), indicating that C228/231 in the RING domain are conserved catalytic sites of RNF115. It has been shown that E3 ubiquitin ligases exert distinct ubiquitin linkage specificity in the context of different E2s. For example, RNF126, a homologue of RNF115, catalyzes K63- and K48-linked polyubiquitin chains in the presence of Ubc13/Uev1a and UbcH5b, respectively (27). However, RNF115 catalyzes K63- and K48-linked polyubiquitin chains in the presence of UbcH5b, whereas RNF115 does not induce ubiquitination in the presence of Ubc13/Uev1a (27), indicating that RNF115 does not preferentially use Ubc13/Uev1a as an E2 enzyme to build diverse ubiquitin linkages.

In an attempt to identify RNF115-binding partners, Amemiya et al. screened the human breast and fetal brain cDNA libraries by using the Bacterio-Match II system and identified polyubiquitin C and ubiquitin A-52 ribosomal fusion protein as RNF115 interactors (23). It is found that the BZF domain of RNF115 binds to ubiquitin with a *K*_d_ of 29.6 (± 3.2) μM, showing a much higher affinity compared with other known ubiquitin-binding domains such as Npl4 zinc finger (NZF) and Mud1 ubiquitin-associated (UBA) domain (23). Mutation of Cys22/25 of RNF115 into A completely diminishes ubiquitin binding but has minimal effects on RNF115-mediated synthesis of K63- and K48-linked polyubiquitin chains (27). These findings demonstrate that RNF115 exerts E3 ligase activity through the C terminal RING domains but independently of its ubiquitin-binding activity through the N terminal BZF domain.

The screening assays with the Bacterio-Match II system also identified UBC9, an E2 for SUMOylation, as an RNF115-interacting protein (23, 28), indicating that RNF115 might be involved in SUMOylation. In support of this notion, it has been reported that RNF115 catalyzes SUMOylation of IκBα in the presence of Ubc9 but not UbcH5, leading to the stabilization of IκBα and inactivation of NF-κB (29). In addition, the RING domain of RNF115 alone could sufficiently inhibit the activation of NF-κB, whereas the RNF115^C228/231A^ fails to SUMOylate IκBα (29), indicating that the N terminal BZF domain is dispensable for SUMOylation activity. Therefore, RNF115 exerts dual ubiquitin and SUMO ligase activities in a manner dependently on its C terminal RING domain in the presence of different E2s.

### The Autoubiquitination of RNF115

It should be noted that RNF115 catalyzes the formation of ubiquitin smear in the above *in vitro* ubiquitination system in which no “substrates” exist (14, 26, 27). It is soon realized that RNF115 itself serves as the substrate and observed that RNF115 catalyzes autoubiquitination within 60 min in the *in vitro* time course ubiquitination assays (14, 20, 23, 26). In addition, mutation of either K26 or K32 into R of RNF115 impaired the autoubiquitination and simultaneous mutation of the two lysine residues completely abolishes the autoubiquitination (23). Interestingly, however, RNF115^K26/32R^ still inhibits NF-κB activity and catalyzes SUMOylation of IκBα (29), indicating that RNF115^K26/32R^ possesses catalytic activity and that K26/32 residues are the autoubiquitination sites of RNF115.

Although RNF115 purified from bacteria catalyzes both K48- and K63-linked polyubiquitin chains *in vitro* (27), the autoubiquitination of RNF115 in cells seems to be K48-linked and susceptible to proteasome-dependent degradation (14). In support of this notion, cycloheximide (CHX) treatment induces rapid degradation of RNF115 that is blocked by the proteasome inhibitor MG132, and RNF115^C228/231A^ fails to induce autoubiquitination or degradation in cells (14, 25). In addition, MG132 treatment increases the spontaneous ubiquitination of RNF115 and leads to the accumulation of RNF115 protein, indicating a constitutive ubiquitination and degradation of RNF115 in cells (23, 25). The basal ubiquitination and degradation of RNF115 are inhibited by RAD23A (also known as hHR23a) and 14-3-3σ in cells, as the expression of RAD23A or 14-3-3σ substantially maintains the levels of RNF115 protein in the presence of CHX (30). Considering RNF115 also serves as a SUMO ligase, it is unknown whether and how RNF115 catalyzes self-SUMOylation in cells and *in vitro*.

### The Transcriptional and Post-Transcriptional Regulation of RNF115

It has been found that RNF115 is highly expressed in breast cancer cells and co-expressed with the estrogen receptor (ER) in more than 70% ER-positive invasive breast ductal carcinomas (16, 17, 28). ERα is a hormone receptor transcription factor that mediates transcription of a large number of genes upon ligand binding. Interestingly, estrogen treatment significantly upregulates the *RNF115* mRNA in a dose-dependent manner in ER-positive T47D and MCF7 breast cancer cells as well as in ER-negative MDA-MB-231 cells stably transfected with ER (28, 31), whereas knockdown of ER substantially impairs the *RNF115* mRNA levels (31). Bioinformatics analyses have identified a potential ER binding site on the promoter of RNF115 gene that is bound to ER after estrogen treatment as revealed by chromatin immunoprecipitation (ChIP)-qPCR assays in MCF7 cells (31). These findings clearly suggest ER functions as an essential transcription factor for *RNF115*.

Besides estrogen, treatment with proinflammatory cytokines such as interleukin 6 (IL-6) and tumor necrosis factor alpha (TNFα) and infection with RNA viruses also induce the upregulation of RNF115 in various cell lines and primary human and mouse cells (25, 29). Though it is implicated that NF-κB binding sites exist in the promoter of *RNF115* gene, knockout of p65, a subunit of NF-κB, has minimal effects on RNA virus-induced upregulation of RNF115 (25), indicating that the classical NF-κB complex is dispensable for transcription of *RNF115*. Because bioinformatics analyses suggest potential AP-1 binding sites in the promoter of *RNF115* gene and AP-1 transcription factors are commonly activated by TNFα and viral infections, it is possible that the upregulation of *RNF115* mRNA is mediated by AP-1 transcription factors downstream proinflammatory cytokines and viral infections.

The levels of *RNF115* mRNA and RNF115 protein are also regulated at post-transcriptional levels. It has been observed that Actinomycin D treatment abolishes the upregulation of *Rnf115* mRNA in primary MEFs after vesicular stomatitis virus (VSV) infection. However, the protein levels of RNF115 are still upregulated by VSV infection or by transfection of poly (I:C) in the presence of Actinomycin D (25). In addition, VSV infection or transfection of poly (I:C) maintains RNF115 protein to a certain level in the presence of CHX (25). These data suggest that RNF115 is regulated at transcriptional, translational, and posttranslational levels after RNA virus infections. However, interestingly, infections with the DNA virus herpes simplex virus 1 (HSV-1) and transfection of dsDNA fail to upregulate or maintain the RNF115 protein or *Rnf115* mRNA in the presence or absence of Actinomycin D or CHX (25), indicating distinct transcriptional and post-transcriptional regulatory mechanisms of RNF115 by RNA and DNA viruses that require further investigations. Several microRNAs have been reported to target *RNF115* mRNA and it is of interest to examine whether such a differential regulation of posttranscriptional regulation of RNF115 depends on the miRNAs (32, 33).

It has been observed that RNF115 is phosphorylated by AKT *in vitro* and sequence analyses suggest a conserved ATK-phosphorylation motif (128-RSRGSS-133) of RNF115 (34). Mutation of S132/133 into A and knockdown or inhibition of AKT activity abrogates AKT1-mediated phosphorylation of RNF115 *in vitro* and in cells (35), suggesting that AKT1 phosphorylates RNF115 on S132/133. Such a phosphorylation leads to the recruitment of 14-3-3 chaperone proteins that inhibit the autoubiquitination and degradation of RNF115 (34). In support of this notion, RNF115^S132/133A^ becomes unstable compared to wild-type RNF115 in cells (35), although RNF115^S132/133A^ and RNF115 are similarly autoubiquitinated *in vitro* (23). In addition, knockdown or inhibition of AKT1 or disruption of RNF115-14-3-3 interactions substantially destabilizes RNF115 protein in various cell lines and primary mouse cells (35, 36). Taken together, these findings suggest a complicated and elegant crosstalk of different post-translational modifications, i.e. phosphorylation of RNF115 by AKT1 inhibits the autoubiquitination and degradation of RNF115 in cells.

The autoubiquitination of RNF115 is also counteracted by deubiquitinase USP9X. It has been recently reported that the deubiquitinase USP9X and RNF115 are correlatively upregulated in breast cancer tissue arrays and breast cancer cell lines (37). USP9X interacts with and counteracts the autoubiquitination of RNF115, thereby maintaining the stability of RNF115. Consistently, knockdown of USP9X leads to increased ubiquitination and degradation of RNF115 in cell lines. Such an observation should be validated *in vivo* with USP9X knockout mice.

### The Subcellular Localization of RNF115

Earlier studies have shown that RNF115 is predominantly located in the cytoplasm in various cell lines (15, 30). Recently, it has been demonstrated that a fraction of RNF115 is located on mitochondria in primary mouse cells as revealed by cell fractionation assays and immunogold staining and electron microscopy analyses, and that the mitochondrial localization of RNF115 is not affected by viral infections (25). More recently, it has been shown that a portion of RNF115 is also colocalized with Calnexin (the ER marker) and GM130 (the Golgi apparatus marker) in immunostaining and confocal microscopy analyses (35), suggesting that RNF115 is located in multiple cellular organelles. In addition, the ER- and Golgi-localization of RNF115 is abolished by knockdown or inhibition of AKT1 or by mutation of S132/133 into A (35), indicating that AKT1-mediated phosphorylation of RNF115 is required for its localization on ER and Golgi apparatus. Interestingly, knockdown or inhibition of AKT1 or mutation of S132/133 into A abolishes the association between RNF115 and 14-3-3 proteins and blocking of 14-3-3 proteins interaction with RNF115 also impairs the ER- and Golgi-localization of RNF115. These biochemical and cellular findings have clearly demonstrated sequential steps of RNF115 localization on the ER and the Golgi apparatus, i.e. AKT1-mediated phosphorylation RNF115 on S132/133 recruits 14-3-3 proteins that escort RNF115 localization on the ER and the Golgi apparatus. It should be noted that the mitochondrial localization of RNF115 is minimally affected by mutation of S132/133 into A, by inhibition of AKT1 or by disruption of 14-3-3-RNF115 associations (35), indicating distinct mechanisms exist for the mitochondrial localization of RNF115. In this context, both RNF115 and RNF115^S132/133A^ interact with RAB7 which has been shown to regulate mitochondria-lysosome contacts (35, 38). Whether and how RNF115 is located in mitochondria dependently on RAB7 require additional investigations.

## The Substrates and Functions of RNF115

To date, more than a dozen of substrates of RNF115 have been identified since its discovery (**Table 1**). By catalyzing ubiquitination or SUMOylation of the substrates, RNF115 has been implicated in various pathways including innate immune signaling, intracellular vesicle trafficking, DNA damage repair, cell cycle and migration and autophagosomal formation.

**Table 1:** Substrates of RNF115.

Target	Types of polyubiquitin chains	Pathways	Refs
RNF115	mixed, E2-dependent		(14, 26, 27)
HIV-1 Gag	unknown		(39)
IκBα	Sumoylation	NF-κB pathways	(29)
MAVS	K48-linked	antiviral signaling	(25)
MITA	K63-linked	antiviral signaling	(25)
EGFR	unknown		(27)
RAB1A/RAB13	K11-linked	trafficking of subcellular compartments	(35)
p21	unknown		(40)
p53	unknown		(41)
APC	unknown		(42)
c-Myc	unknown		(43)

### RNF115 in Antiviral Immunity

**Figure 2:**
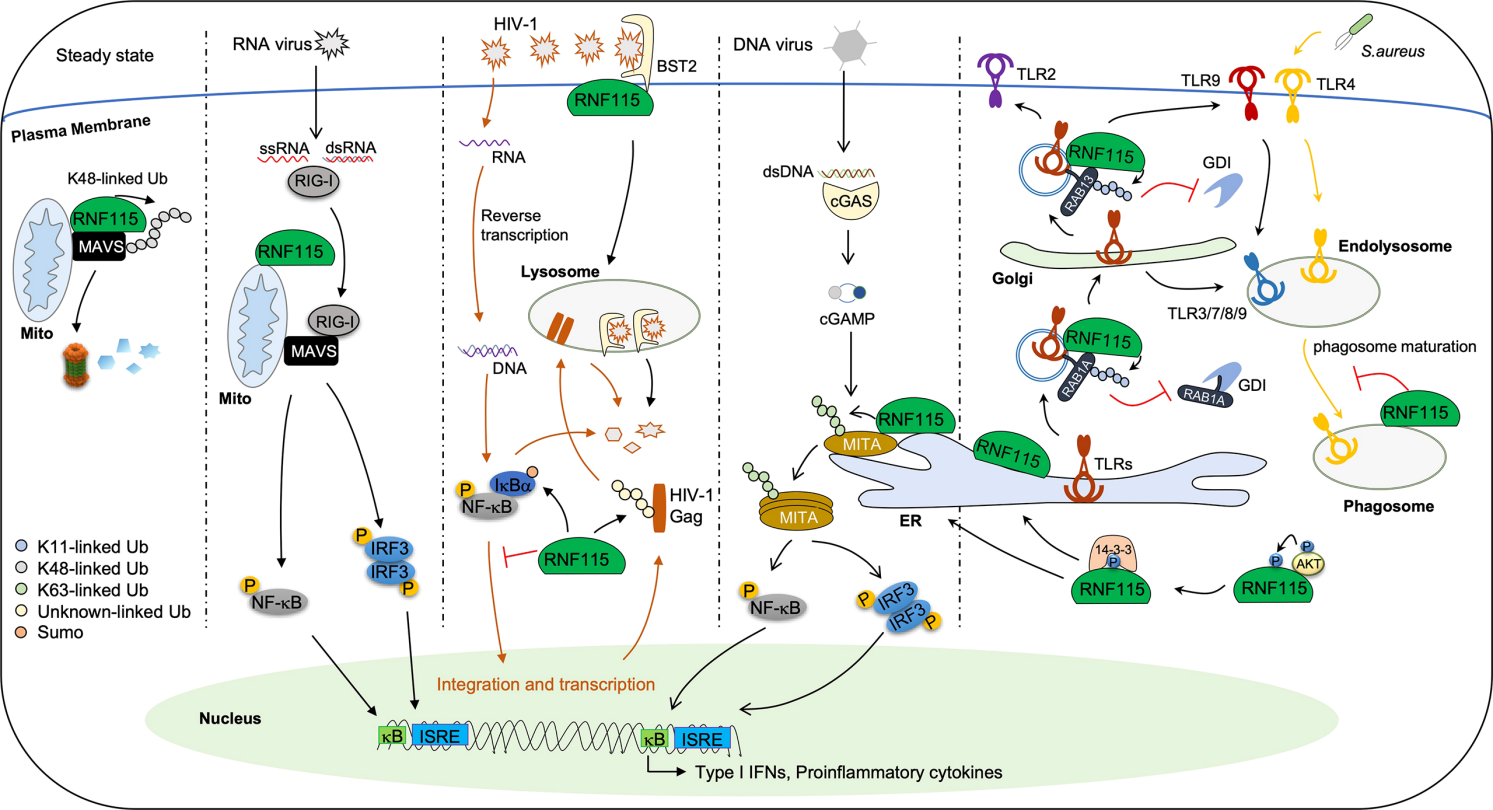
The signaling pathways regulated by RNF115 in innate immunity. In steady state, RNF115 constitutively interacts with and catalyzes K48-linked ubiquitination of MAVS, leading to the proteasomal degradation of homeostatic MAVS. Upon infection with RNA viruses, RIG-I–MAVS interaction led to RNF115 dissociation from MAVS, thereby initiating downstream signal transduction. Moreover, RNF115 functions as a restriction factor for HIV-1 infection. Firstly, RNF115 interacts with BST2 to promote the newly synthesized HIV-1 particles on the plasma membrane for lysosomal degradation. Secondly, RNF115 directly catalyzes ubiquitination of HIV-1 Gag, leading to its lysosomal degradation independently on BST2. Thirdly, RNF115 targets IκBα for SUMOylation, inhibiting HIV-1-induced NF-κB activation and HIV-1 transcription. In addition, The 14-3-3 chaperones bind to AKT1-phosphorylated RNF115 and facilitate RNF115 localizing at the ER. RNF115 interacts with and induces K63-linked ubiquitination of MITA at ER during infection of DNA viruses, promoting aggregation of MITA and the production of type I IFNs and proinflammatory cytokines. RNF115 also interacts with TLRs and buds off the ER onto the coat protein complex II vesicles. RNF115 catalyzes K11-linked ubiquitination of RAB1A to inhibit the extraction of RAB1A by GDI1. In addition, RNF115 inhibits the post-Golgi trafficking of TLR2, TLR4, and TLR9 by catalyzing K11-linked ubiquitination on RAB13. Consequently, the trafficking of TLR4 from the Golgi apparatus to cell surface and the trafficking of TLR9 to cell surface en route to endolysosomes were inhibited. Finally, RNF115 also inhibits phagosome maturation during *S. aureus* infection.

Several studies have suggested that RNF115 is a restriction factor for HIV-1 infection (**Figure 2**). It has been found that RNF115 interacts with tetherin to facilitate the internalization of newly synthesized HIV-1 particles on plasma membrane for lysosomal degradation, thereby preventing viral release from the infected cells (44). However, it is unclear whether RNF115 functions dependently on its enzymatic activity or whether RNF115 catalyzes the ubiquitination of tetherin. Later, it has been shown that RNF115 directly induces ubiquitination of HIV-1 Gag and targets it for lysosomal degradation in a manner dependent on its catalytic activity but independently of tetherin (39). More recently, two reports have demonstrated that RNF115 inhibits HIV-1-induced activation of NF-κB to restrict transcription of HIV-1 by targeting IκBα for SUMOylation (29, 45). These findings collectively indicate that RNF115 biochemically targets both viral and host factors to restrict HIV-1 infection in cultured cells with an *in vitro* overexpressed system, and thus further investigations are required to examine the role of RNF115 in HIV-1 infection *in vivo* under physiological conditions.

A recent study has shown that RNF115 plays dual roles in innate immune responses against RNA and DNA viruses (25) (**Figure 2**). RNF115 interacts with the adaptor protein MAVS constitutively on mitochondria and induces K48-linked ubiquitination and proteasomal degradation of MAVS. VSV infection activates RIG-I that competitively binds to MAVS and thereby impairs RNF115-MAVS associations. Therefore, knockout of RNF115 results in accumulation of MAVS and hyper-resistance to RNA virus infections. In contrast, RNF115 interacts with another adaptor protein MITA to catalyze K63-linked ubiquitination of MITA on the ER after HSV-1 infection. Such a modification promotes the oligomerization of MITA and the recruitment of TBK1 and IRF3. As a result, deficiency of RNF115 leads to hyper-sensitivity to DNA virus infections. It should be noted that several RNA viruses activate MITA through various mechanisms and that VSV or SeV infections increase the ER localization of RNF115 (46–48). Thus, it is likely that RNF115 keeps MAVS in check under homeostatic conditions by catalyzing K48-linked ubiquitination of MAVS and promotes cellular antiviral responses after RNA virus infections by catalyzing K63-linked ubiquitination of MITA, respectively. Though it is observed that RNF115 constitutively exerts K48 linkage specificity on mitochondria, while HSV-1 infection somehow alters the ubiquitin linkage specificity of RNF115 by changing its location from mitochondria to ER, so far it is unclear how RNF115 exerts different ubiquitin linkage activities on different substrates or at distinct subcellular organelles. In addition, considering that MITA plays essential roles in self DNA-induced autoimmunity and tumor immunity (49), it is of great interest to examine whether and how RNF115 regulates autoimmunity and tumorigenesis in the future.

### RNF115 in Cell Migration, Growth and Death

Because RNF115 is upregulated in breast cancer cells and tissues and negatively correlated with lymph nodes metastasis and disease-free survival for regional recurrence (14), the roles of RNF115 in cell growth and migration have been intensively investigated (**Table 2**). It has been shown that overexpression of RNF115 or RNF115^C22/25A^ but not RNF115^C228/231A^ significantly promotes the growth, proliferation and migration of NIH3T3, T47D and MCF7 cells (14, 23), whereas knockdown of RNF115 has an opposite effect, indicating a pro-growth role of RNF115 in cell lines dependently on its enzymatic activity. Later, it has been reported that RNF115 promotes DNA damage repair caused by ultraviolet light *via* interaction with ATM, γH2AX and Rad51. Consequently, knockdown of RNF115 increases DNA damage and leads to growth arrest of cell lines (50). It has been further observed that overexpression of RNF115 but not RNF115^S132/133A^ or RNF115^C228/231A^ inhibits and knockdown of RNF115 promotes the phosphorylation and activation of AMPKα1, respectively (36), indicating that both the enzymatic activity and the AKT-mediated phosphorylation of RNF115 are required for AMPK activation. In addition, knockdown of RNF115 or inhibition of AKT sensitizes metformin-mediated growth inhibition of multiple breast cancer cell lines. These observations are in agreement with the findings that RNF115 phosphorylation on S132/133 by AKT induces the ER, Golgi and endolysosome localization of RNF115 and AMPK functions at different subcellular compartments such as lysosome and ER for glucose sensing and energy metabolism (35, 53, 54). However, because RNF115 does not induce the ubiquitination of AMPKα1, the direct target of RNF115 for the regulation of AMPKα1 phosphorylation and activation remains to be identified.

**Table 2:** The functional roles of RNF115 in cancer.

Cancer type	Function	Mechanism	Refs
Breast Cancer	promote cancer growth,proliferation and migration; promote DNA damage repair	interaction with ATM, γH2AX and Rad51; inhibition the phosphorylation and activation of AMPKα1; promotation ubiquitination and degradation of p21; reduction lysosomal degradation of EGFR and increase EGFR phosphorylation	(14, 23, 36, 40, 50, 51)
Lung adenocarcinoma	promotation growth and inhibition the apoptosis of cancer cell	catalyzation the ubiquitination of p53; catalyzation the ubiquitination of APC to modulate the Wnt/β-catenin activation	(41, 42)
Lung cancer	inhibition cancer cell growth	induction ubiquitination and degradation of cMyc	(43)
Gastric cancer	promotation cancer cell growth	interaction and stablization SNX7 to promote autophagosome maturation	(52)

A lot of efforts have been made to identify the substrates of RNF115 for the regulation of cell growth and viability. It has been shown that overexpression of RNF115 interacts with ectopically expressed EGFR in a c-Cbl-dependent manner and catalyzes ubiquitination of and degradation of EGFR (26, 27). However, overexpression of RNF115 in cells reduces lysosomal degradation of endogenous EGFR after EGF stimulation which increases the duration of EGF signaling and EGFR phosphorylation in HeLa cells (51). In addition, RNF115 directly interacts with and promotes ubiquitination and degradation of p21 and knockdown of p21 partially rescues cell growth arrest caused by knockdown of RNF115 in ER^+^ breast cancer cells (40). In lung adenocarcinoma cell lines, RNF115 has been shown to catalyze the ubiquitination of p53 and knockdown of RNF115 significantly inhibits the cell viability *in vitro* by inducing G1 phase arrest and tumor growth in a xenograft model (41). Moreover, RNF115 overexpression promotes ubiquitination of adenomatous polyposis coli (APC) to modulate the Wnt/β-catenin pathway activation, and thereby promotes the proliferation and inhibits the apoptosis of lung adenocarcinoma cell lines (42). In contrast, however, another study has reported that RNF115 induces ubiquitination and degradation of c-Myc and thereby inhibits H1299 lung cancer cell growth (43). The reason behind the discrepancies remains unknown. It should be noted that all the experiments have been carried out with cell lines and xenograft models and that knockout of RNF115 does not affect the breeding of mice and the growth and development of mice and various types of cells (25). Therefore, the effects of RNF115 on cell migration, growth and death should be re-evaluated *in vivo* under physiological or pathological conditions.

### RNF115 in Intracellular Trafficking and Autophagosomal Maturation

One of the earliest studies on RNF115 has characterized RNF115 as a RAB7-interacting protein (15). The RAB proteins are small GTPases that play essential roles in intracellular vesicle trafficking (55–57). Specifically, RAB7 is involved in late endocytic traffic and lysosome acidification as well as mitochondria-lysosome contact (38). It is observed that overexpression of RNF115 promotes the late endosome-lysosome fusion and acidification and the trafficking of HIV-1 viral particles to the lysosomes for degradation (15, 39, 44), whereas knockdown of RNF115 leads to the retention of EGFR in the late endocytic compartments and hyper-phosphorylation and stabilization of EGFR (27). However, it is unlikely that RNF115 regulates the endocytic trafficking or acidity *via* RAB7, as RNF115 does not induce ubiquitination of RAB7 (26). Instead, knockdown of RNF115 results in downregulation of Vps22, a component in the endosomal sorting complexes required for transport (ESCRT) II machinery (27), which might be responsible for the inhibition of the fusion of endosomes (or multivesicular body) and lysosomes. Interestingly, a recent study has reported that knockdown of RNF115 destabilizes SNX7, a component in the SNARE complex for membrane fusion of subcellular compartments, leads to elevation of endogenous LC3B-II protein and inhibits the fusion of autophagosomes with lysosomes in multiple cell lines (52). It should be noted that RNF115 ubiquitinates neither Vps22 nor SNX7, indicating an indirect regulation of Vps22 and SNX7 for autophagosome maturation and endosome-lysosome fusion by RNF115. Although the mechanisms are incompletely clear, these studies strongly suggest that depletion of RNF115 promotes the formation and movement of subcellular compartments possibly by blocking their fusion with lysosomes in cell lines.

It is until recently that the *in vivo* roles of RNF115 in the trafficking of subcellular compartments have been intensively studied. In an unbiased identification of proteins on IFNγ-induced phagosomes, Bilkei-Gorzo et al. have found that RNF115 and K63/48/11-linked polyubiquitin chains are significantly enriched on the phagosomes (58) (**Figure 2**). They further show that knockout of RNF115 promotes phagosome maturation and acidification in primary mouse macrophages under homeostatic conditions. Interestingly, quantitative proteomics data suggest that the ER and the peroxisome proteins are reduced in the phagosomes from RNF115 deficient macrophages compared to the wild-type macrophages, and such a reduction is fully rescued by reconstitution of RNF115 but not RNF115^C228/231A^ into RNF115 KO macrophages (58). These data suggest an inhibitory role of RNF115 in phagosome maturation in primary mouse macrophages dependently on its E3 ligase activity, though the substrates of RNF115 remain to be characterized. More recently, it has been demonstrated that RNF115 catalyzes K11-linked ubiquitination of RAB1A and RAB13 to inhibit their association with guanosine diphosphate (GDP) dissociation inhibitor (GDI) for re-activation, thereby negatively modulating the trafficking from ER to Golgi apparatus and from Golgi apparatus to cell membrane in primary macrophages, dendritic cells and lung fibroblasts, respectively (35) (**Figure 2**). Consistently, knockout of RNF115 promotes the trafficking of TLRs from ER to Golgi apparatus and to lysosomes and cell membrane. Therefore, the RNF115 knockout mice are more resistant to bacterial infections and sensitive to imiquimod-induced autoimmunity (35, 58). In contrast, knockout of RNF115 does not affect the endocytosis of carboxylated particles or TLRs after ligands stimulation (35, 58). It should be noted that TNF- or IL-1β-induced secretion of CXCL1 or IL-6 is not affected by knockout of RNF115 and that the expression of MHC-I and the integrins CD11b or CD11c on cell surface through post-ER trafficking is comparable between wild-type and RNF115 deficient BMDCs (35), indicating that not all the trafficking of subcellular compartments such as the secretory pathway is regulated by RNF115. Collectively, these studies have revealed an essential role of RNF115 in inhibiting the post-ER trafficking of subcellular compartments by targeting non-degradative ubiquitination of RAB proteins *in vivo*.

## Prognosis and Therapeutic Values of RNF115 in Diseases

RNF115 is highly expressed in invasive breast cancer and the levels of RNF115 are positively associated with ER levels in the tissue arrays of breast cancer (14, 28). Large scale genome wide-associated studies have identified two variants rs12405132 and rs17354678 at 1q21.1 where *RNF115* is located as new susceptibility loci for breast cancer, though it is unclear how the variants affect *RNF115* or other genes for the progression of breast cancer development (18, 32). Analyses with the bulk-seq database suggest that high expression of *RNF115* is correlated with poor prognosis in gastric, ovarian and breast cancers, adrenocortical carcinoma, acute myeloid leukemia and kidney chromophobe (51, 59). In contrast, there are discrepancies for the roles of RNF115 in lung cancer, as high *RNF115* mRNA levels are associated with an improvement in overall survival of lung cancer and poor overall survival of lung adenocarcinoma (41, 51). The reason behind the discrepancies is unclear. However, it has been shown that RNF115 promotes lung adenocarcinoma by targeting p53, APC and c-Myc for ubiquitination and degradation, indicating complicated roles of RNF115 in lung cancer (41–43). RNF115 is expressed in all renal oncocytoma cases and in cases designated as oncocytic neoplasm which favor oncocytoma but barely expressed in renal cell carcinoma (60). These findings suggest RNF115 as a prognostic factor for different types of cancers.

By analyzing rare copy number variants in heterotaxy syndrome patients with congenital heart defects, Liu et al., have found that genic deletion of RNF115 is a strong candidate associated with this disease (61). They further show that knockdown of *rnf115* in zebrafish by morpholino oligos increases abnormal cardiac looping that is rescued by injection of *rnf115* mRNA in the embryos. However, deletion of RNF115 in mice does not lead to obvious abnormalities including breeding, growth and development as long as one year without any challenges (25). Bioinformatics analysis suggests that RNF115 may be connected with the occurrence and development of atherosclerosis (62), which waits for further investigations in cellular and animal disease models.

## Conclusive Remarks and Perspectives

Since the discovery of RNF115 about two decades ago, the roles of RNF115 in various signaling pathways and disease progression have been emerging. Though most of the studies on RNF115 have been performed in cell lines *in vitro*, they have substantially advanced our understanding of RNF115. In the future, more efforts should be made to investigate the *in vivo* functions and substrates of RNF115 in depth with animal models. The first step would be to systemically identify the differentially ubiquitinated proteins or peptides in wild-type and RNF115 knockout cells or tissues and then to biochemically examine whether RNF115 catalyzes ubiquitination of the proteins. In addition, it should be noted that there are the different isoforms of RNF115 in cells and all of the studies on RNF115 choose to study the longest one. Therefore, whether and how different RNF115 isoforms compensate each other to play similar or distinct functions require further investigations. RNF115 is an E3 ligase and most of the RNF115 functions depend on its ligase activity. It is conceivable to develop inhibitors for RNF115 to treat diseases and modulate pathways potentially regulated by RNF115 (63). Because of the multifaceted roles of RNF115 in various pathways by targeting different substrates, it should be careful to treat diseases with RNF115 as a target. For example, RNF115 negatively regulates TLRs-mediated signaling and autoimmunity and targeting RNF115 might promote TLR4-related sepsis or TLR7/9-related autoimmunity. In summary, systemic consideration of the multifaceted roles of RNF115 should be taken when exploring the *in vivo* roles of RNF115.

## Author Contributions

M-XW wrote this manuscript. Z-DZ and TL revised this manuscript. All authors contributed to the article and approved the submitted version.

## Funding

This study was supported by grants from the Natural Science Foundation of China (Grant No. 32000636) and the Fundamental Research Funds for the Central Universities (Grant No. 2042022kf1123).

## Conflict of Interest

The authors declare that the research was conducted in the absence of any commercial or financial relationships that could be construed as a potential conflict of interest.

## Publisher’s Note

All claims expressed in this article are solely those of the authors and do not necessarily represent those of their affiliated organizations, or those of the publisher, the editors and the reviewers. Any product that may be evaluated in this article, or claim that may be made by its manufacturer, is not guaranteed or endorsed by the publisher.
